# Myocardial strain analysis with CMR in cardiotoxicity patients using deformation field analysis: Comparison to healthy volunteers and heart transplant patients

**DOI:** 10.1186/1532-429X-18-S1-W30

**Published:** 2016-01-27

**Authors:** Abraham Bogachkov, Kai Lin, Benjamin H Freed, Michael Markl, James C Carr, Jeremy D Collins

**Affiliations:** 1 grid.465264.7Feinberg School of Medicine, Northwestern University, Chicago, IL USA; 2 Cardiovascular MR R&D, Siemens Healthcare, Chicago, IL USA; 3 grid.465264.7Cardiology, Northwestern University, Chicago, IL USA; 4 grid.465264.7Radiology, Northwestern University, Chicago, IL USA

## Background

Noninvasive monitoring of cardiac function in patients with iatrogenic or environmentally-induced cardiotoxicity is of prime importance as a measure of disease severity. Strain imaging at cardiac MR (CMR) has been previously shown to be a highly robust modality in the detection of early cardiac dysfunction in the heart failure population. However, strain analysis has not previously been applied in the assessment of cardiotoxicity patients. The purpose of this study is to compare radial and circumferential LV strain values in patients with known cardiotoxicity to healthy volunteers and post-heart transplant patients, and to then correlate the cardiotoxicity strain values to calculated LV ejection fractions.

## Methods

Retrospective analysis of CMR images from 11 volunteers (9/11 males, avg. age 51.6), 5 heart transplant patients (5/5 males, avg. age 60.2), and 10 cardiotoxicity patients (3/10 males, avg. age 48.9) were obtained at 1.5 T (MAGNETOM Avanto, Siemens Medical Systems, Erlangen, AG) using GRAPPA factor 2 acceleration. Myocardial strain analysis at CMR was performed using semi-automatic prototype software calculating Lagrangian strain from deformation field analysis (Siemens Corp, Corporate Technology, Princeton, NJ). Left ventricular (LV) midwall average and peak systolic radial and circumferential strains data was calculated. Strain data between groups was compared using univariate analysis of variance (ANOVA) to assess statistical equivalence. Cardiotoxicity patients' strain data was compared to calculated LVEF values using linear regression with associated R^2^ values.

## Results

**Figure 1:**
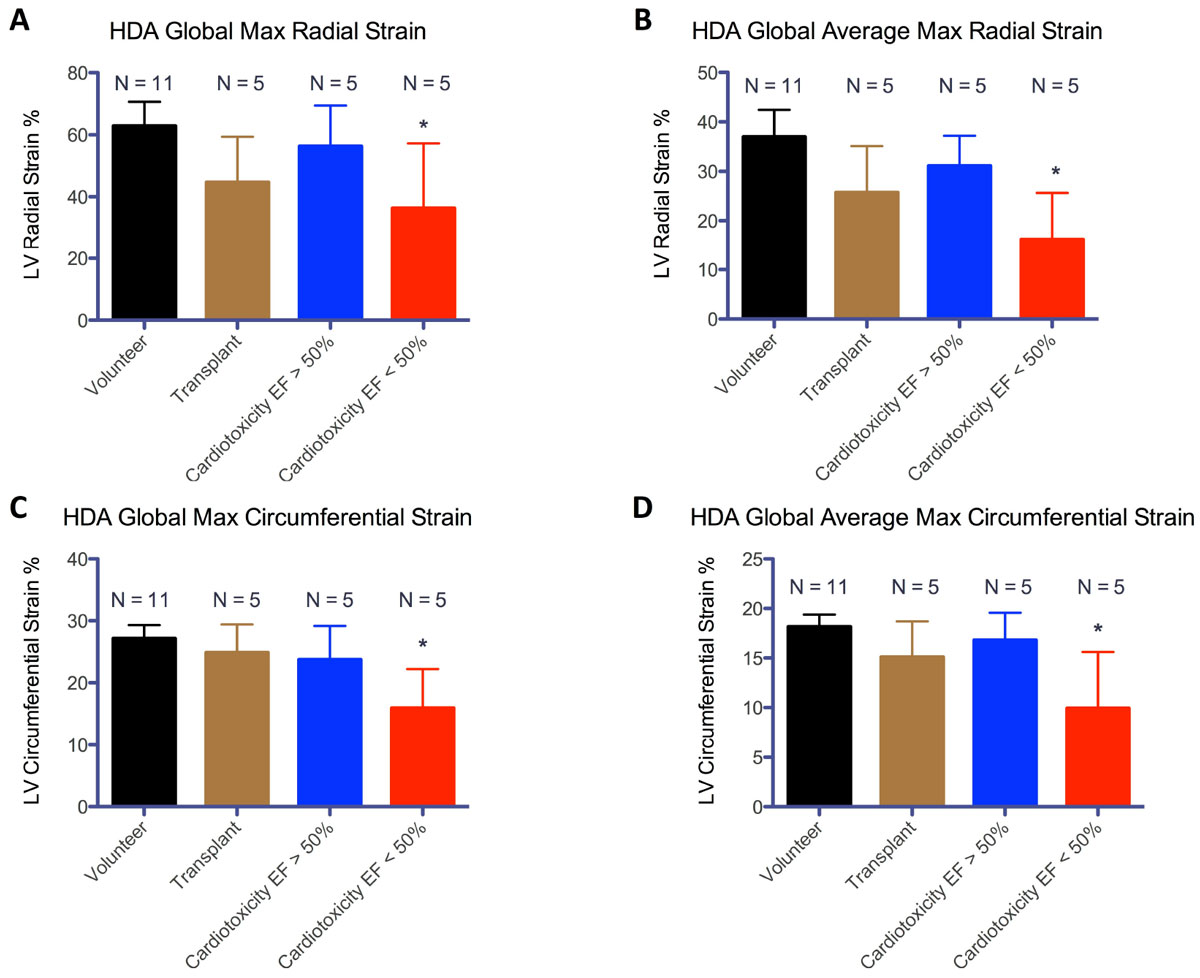
**Average left ventricular strain as calculated by a semi-automatic prototype software calculating Lagrangian strain from deformation field analysis with 95% CI**. **A** - mean peak radial strain value amongst the 3 groups; **B** - mean of average segmental peak radial strain value amongst the 3 groups; **C** - mean peak circumferential strain value amongst the 3 groups; **D** - mean of average segmental peak circumferential strain value amongst the 3 groups. * indicated statistically significant (p < 0.05) difference from volunteer group.

**Figure 2:**
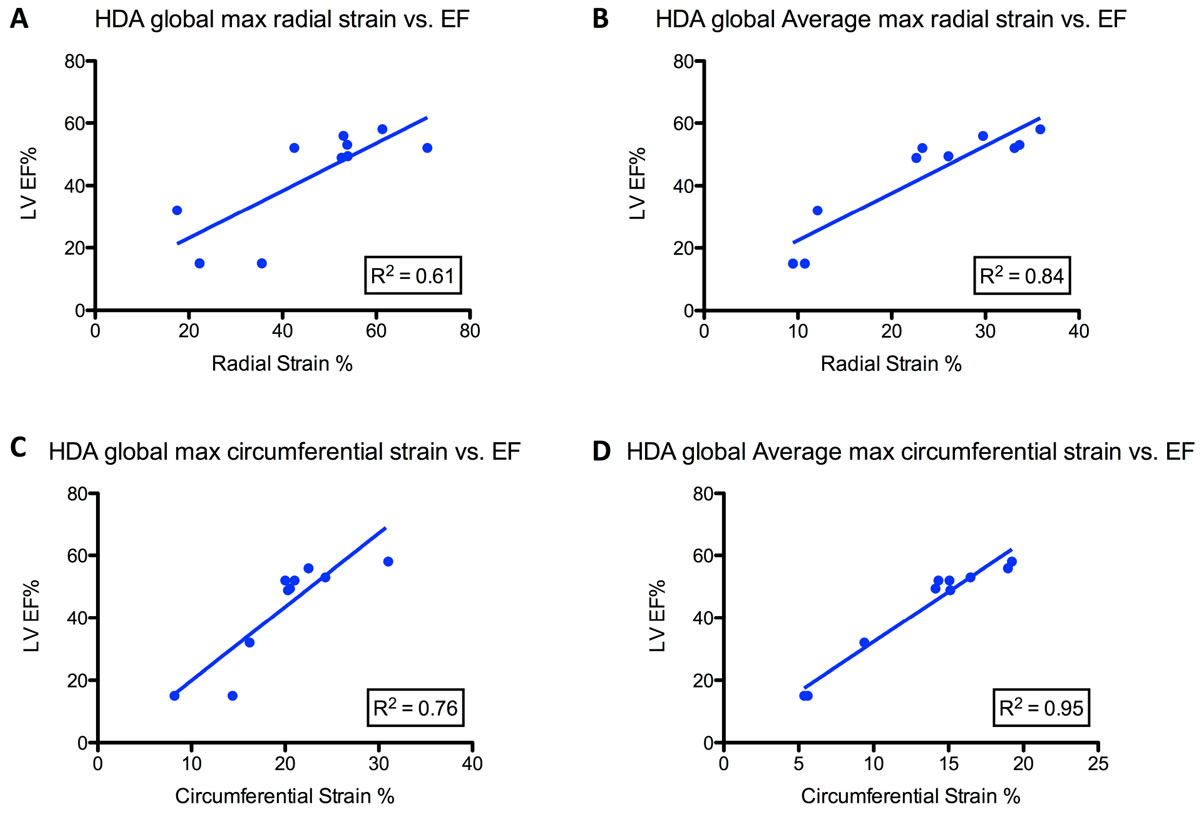
**A - linear regression of mean peak radial strain value from the cardiotoxicity group vs. calculated LV EF**; **B** - linear regression of mean of average segmental peak radial strain values from the cardiotoxicity group vs. calculated LV EF; **C** - linear regression of mean peak circumferential strain value from the cardiotoxicity group vs. calculated LV EF; **D** - linear regression of mean of average segmental peak circumferential strain values from the cardiotoxicity group vs. calculated LV EF.

Mean global peak radial and circumferential strain values and mean average-segmental peak radial and circumferential strain values were calculated for subjects in the 3 cohorts. Mean strain values were found to be lower in cardiotoxicity patients with EF < 50% vs. healthy volunteers in both peak radial and circumferential strain in a statistically significant (p < 0.05) manner (Figure 1). All other groups analyzed were found to have statistically equivalent mean strain values, including both subsets of cardiotoxicity patients. The R^2^ values for linear regression of global peak radial and circumferential strain values vs. EF were 0.61 and 0.76, and 0.84 and 0.95 for mean average-segmental peak radial and circumferential strain, respectively (Figure 2).

## Conclusions

Radial and circumferential LV strain values in patients with known cardiotoxicity were found to be lower than volunteers in a statistically significant manner when using a semi-automatic prototype software calculating Lagrangian strain from deformation field analysis. Furthermore, strain values were found to correlate strongly with LVEF, a well-established clinical marker of cardiotoxicity, particularly in the mean average-segmental peak radial and circumferential strain values. Continued work is necessary to elucidate a more defined clinical role for CMR-calculated strain in the cardiotoxicity patient population.

